# Periscope of Dermatology

**Published:** 1897-11-27

**Authors:** 


### PERISCOPE OF DERMATOLOGY.

(*Continued from page* 139.)

**Eczema.**—Shoemaker[14] gives extract hamamelidis virg. in cases of chronic eczema to contract the vessels. A teaspoonful of this drug mixed with equal parts of glycerine is given three times a day. He recommends the following ointment as useful in allaying itching, and in stimulating the parts: ℞. acid carbolic ♏ xv.; acid salicylici, gr. xv.; sulphur sublim., grs. xx.; ung. zinci oxidi, ung. aq. rosæ, aa ʒ iv. ♏. ft. ungt. In facial eczema of infants the crusts must first be removed with a poultice, and the following ointment applied: ℞. pulv. marantæ, ʒ js.; zinc carbonates, ʒ ijs.; ung. aq. rosæ, js., ♏. ft. ungt.

Duhring[15] discusses fully the local treatment of eczema, with regard to the regions in which it occurs, referring to the methods which have given the best results in his practice. He is distinctly in favour of tar preparations, and recommends a weak preparation to begin with, the strength of which is gradually increased. In that most troublesome complaint, eczema of the anus, he has given much ease by the following ointment: ℞. sulph. præcipitat., gr. 40; naphthol, gr. 20; morphine, gr. ii.; zinci carbonat., ʒ i; cold cream, ℥ i. ♏. He advises the use of cold cream as a basis to the ointment, since it is more cooling than vaseline.

Colcott Fox,[16] although formerly believing in the constitutional causation of infantile eczema, is now inclined to believe that the condition is probably parasitic, since it nearly always commences in the head and spreads downwards. The following are the chief indications in treatment: (1) Cleanliness; (2) the application of local remedies, first soothing, then parasiticides; (3) the prevention of scratching and rubbing; and (4) the correction of any intercurrent discidu.

Pusey,[17] on the other hand, is inclined to regard infantile eczema not a parasitic, but as due to toxæmia due to: (1) Absorption from the intestinal canal; (2) absorption from the mother's milk; and (3) defects in metabolism, or to a failure in the balance between assimilation and excretion due to too much or too rich feeding. He therefore lays greater stress on correcting the general condition than on applying antiparasitic ointments.

Brelkley[18] also relies mostly on constitutional treatment (including diet and hygiene) in cases of infantile eczema, and by these means alone a recurrence can be prevented. Protective and soothing applications should be used locally, the best of which is zinc oxide ointment.

Cantrell[19] describes the special appearance of eczema when it affects the umbilicus. The disease starts as an intertrigo, the sulci alone being affected at first. Crusts forms on these, and so prevent the escape of discharge, which accumulates in the navel and decomposes, producing a disagreeable looking and smelling place. Considerable induration follows, and inflammation and excoriation extends some way round the umbilicus. When there is much swelling, boracic fomentations should be applied first, and when the swelling subsides a zinc ointment, containing ten to forty grains to the ℥i of calomel or acetanelid, should be employed. Should this fail, more stimulating ointments, such as those of resorcin, salicylic acid, or salol, should be tried. The most troublesome cases are those when the disease has been confined to the umbilical depression for some time; in these cases nitrate of silver, in solution of ten to twenty grains in one ounce of oil, can be applied, but its use is painful.

Equally efficacious, but less painful, is an ointment of resorcin or salicylic acid in the strength of 40 grams to 2 or 3 ʒs. of vaseline. This is kept in close contact with the sore for two or three hours at a time. Its use is then followed by a mild astringent ointment.

Schubert[20] described the case of a lady, aged 30, who for seventeen years suffered from general moist eczema, for which every kind of treatment had been tried in vain. After the first venesection the eczema dried up, and four weeks after the third venesection the patient was cured. Schubert found in blood obtained by venesection that there was an increase in the number of white corpuscles in skin diseases, just as in any other illnesses. The accumulation of white corpuscles in the cutaneous capillaries offers a good nutrient material for the growth of parasitic microbes, which excite skin diseases. His explanation of the good effect of bleeding in some cases of eczema is that with each venesection the blood becomes poorer in these white corpuscles. Improvement was also noticed in cases of psoriasis and of furuncolosis.

14 Med. Bull., July, 1897, p. 247. 15 Amer. Journ. Med. Sci., April, 1897, p. 379. 16 Treatment, vol. ii., p. 55. 17 Chicago Clin. Rev., Sept., 1897, p. 607, 18 Treatment, vol. ii., p. 61. 19 Ther. Gaz., Feb. 15, 1897, p. 82. 20 Berlin, Klin. Woch., 1897, No. 16; B. M. J. Ep., May 15, 1897 (No. 387).

